# Mosaicism for structural non-centromeric autosomal rearrangement in prenatal diagnoses: evidence for sex-specific selection against chromosomal abnormalities

**DOI:** 10.1186/s13039-017-0346-0

**Published:** 2017-12-11

**Authors:** Natalia V. Kovaleva, Philip D. Cotter

**Affiliations:** 1 Academy of Molecular Medicine, Mytniskaya str. 12/44, St. Petersburg, Russian Federation; 2 Department of Pediatrics, University of California San Francisco, San Francisco, CA USA; 3 ResearchDx Inc., Irvine, CA USA

**Keywords:** Segmental somatic mosaicism, Non-centromeric autosomal rearrangement, Genomic imbalance, Sex ratio, Maternal age, Paternal age

## Abstract

**Background:**

Mosaicism for chromosome rearrangements is common in preimplantation diagnoses, yet is rare in prenatal diagnoses as well as in other groups of patients referred to cytogenetic testing. Consequently, there is a lack of detailed studies on this kind of mosaicism in all groups of patients. Previous reports have identified a deficit of males among asymptomatic carriers of N/unbalanced Rea. Three mechanisms were proposed for explaining this phenomenon, including a high instability in the early female embryonic development, a male-specific selection against abnormal cells in the early embryo development, or a high intrauterine lethality of male carriers. To address these possibilities, we have performed a meta-analysis of male-to-female ratio (sex ratio, SR) in prenatally diagnosed and in spontaneously aborted carriers of mosaic Rea.

**Results:**

One hundred and twenty one prenatally detected cases of normal cell line/autosome rearrangement mosaicism (N/Rea) with known carriers’ sex were identified from the literature. Carriers of N/unbalanced Rea presented with 38 abnormal and 28 normal/apparently normal outcomes while carriers of N/balanced Rea presented with 24 normal and 3 abnormal outcomes. 58% of carriers of N/unbalanced Rea with an abnormal outcome displayed a high proportion (> 50%) of amniocytes with the abnormality compared to 25% of carriers with normal/apparently normal outcome. More female carriers of N/unbalanced Rea were identified with an abnormal outcome (15 M/23F) in contrast to a notable male predominance (18 M/10F) among those with normal outcome*.* Additionally, among spontaneously aborted carriers of N/unbalanced Rea, there was a strong female predominance (7 M/23F).

**Conclusion:**

Previous reports have identified a deficit of male among asymptomatic carriers of N/unbalanced Rea. The current data suggests a male-specific selection against chromosomal abnormalities.

**Electronic supplementary material:**

The online version of this article (10.1186/s13039-017-0346-0) contains supplementary material, which is available to authorized users.

## Background

Mosaicism is the presence of more than one genetically distinct cell line in a single organism that originate from a genetically homogenous zygote. Mosaicism may cause a variety of clinical problems including clinical manifestations of genomic imbalance (growth and/or developmental delay, congenital malformations, cognitive disability, neurological impairment, pigmentation anomalies, etc.), a high risk of affected progeny, and reproductive problems (infertility, habitual miscarriages).

Mosaicism is astonishingly frequent in early human development. However, it is rarely found in later stages, because of effective selection against abnormal cells (self-correction) or due to mitotic arrest and early wastage of abnormal embryos [1–3]. Segmental mosaicism, i.e. mosaicism for structural chromosomal rearrangement (Rea), is not as frequent as mosaicism for whole chromosomes, comprising about 15% of all detected cases [3]. Carriers of segmental mosaicism are also rarely found both among prenatal diagnoses [4] and among patients referred for cytogenetic testing [5]. In the general population, segmental mosaicism for balanced chromosome rearrangements (balanced translocations and inversions) and unbalanced autosome rearrangements is extremally rare, with calculated frequencies of 0.02 ‰, 0.005 ‰ respectively; segmental mosaicism for unbalanced autosomal rearrangements was reported as 0.002 ‰ [6].

A remarkable feature of mosaicism for unbalanced Rea is a strong prevalence of females among asymptomatic carriers, in contrast to carriers of mosaicism for balanced rearrangements with a typical slight male prevalence [6]. Among affected carriers of mosaicism for unbalanced Rea there is pronounced female preponderance. In contrast, there is no sex bias among carriers of mosaicism for balanced rearrangements [7].

Three mechanisms were suggested for explaining the female predominance among carriers of mosaicism for unbalanced Rea, including a high intrauterine lethality of male carriers, a male-specific selection against abnormal cells in the early embryo development, or a high instability in the early female embryonic development [6, 7]. High instability in early female embryonic development would predict a female prevalence among both carriers of balanced and unbalanced Rea, arguing against this mechanism.

To address the other two possibilities, we have performed an analysis of male-to-female ratio (sex ratio, SR) in prenatally diagnosed and in spontaneously aborted carriers of mosaic Rea. A male-specific selection against abnormal cells would result in a higher proportion of males among normal pregnancy outcomes along with a higher proportion of females among abnormal outcomes. A high intrauterine lethality of male carriers of mosaicism for unbalanced Rea would result in a strong preponderance of males among abortuses. Therefore, the objectives of the present study were: (i) determination of the outcome of pregnancies where mosaicism for Rea was detected prenatally, (ii) analysis of the sex ratio according to pregnancy outcomes and type of rearrangement, (iii) analysis of the sex ratio among spontaneously aborted carriers of mosaic Rea.

## Methods

We reviewed reports in the literature on carriers of normal line/rearrangement (N/Rea) mosaicism detectable either by conventional cytogenetics or by molecular cytogenetics. The cases were identified from various sources including PubMed. Only reports of mosaicism N/Rea carriers of known sex were selected for the study. We excluded cases of Rea with both breakpoints localized at pericentromeric regions, because of the strong female preponderance among carriers of such mosaicism [8, 9]. One hundred and twenty one cases of prenatally detected carriers of N/Rea, along with the data on their chromosome constitution, parental ages, proportion of abnormal cell line(s), and the indication for testing have been identified (listed in Additional file 1: Table S1, Additional file 2: Table S2, Additional file 3: Table S3, Additional file 4: Table S4, Additional file 5: Table S5, Additional file 6: Table S6 and Additional file 7: Table S7 are listed in the Additional file 8: Supplemental References). They were subdivided as normal/apparently normal and abnormal. Additionally, data on 29 mosaic fetuses miscarried/aborted spontaneously were retrieved from the literature (Additional file 7: Table S7). Data were analyzed using standard statistics, a Chi-square test with Yates correction. The comparison of observed and expected proportions was made using binomial test.

## Results and discussion

### N/Rea profile

In the sample of prenatal cases evaluated, deletions were present in 34 (28%), duplications in 12 (10%), rings in 14 (12%), derivative chromosomes in 11 (9%), other Reas in 17 (14%), and apparently balanced Rea in 33 (27%) of the cases. The proportion of carriers of balanced Rea was different from that found either in asymptomatic carriers (51%) or in affected carriers diagnosed postnatally (5%) (see Table 1 presented published data on asymptomatic and affected postnatally diagnosed carriers). The profile of 88 cases of unbalanced Reas was: 39% deletions, 14% duplications, 16% rings, 12% unbalanced translocations, and 19% other Rea. There are some differences in N/rea profile between prenatally diagnosed carriers and both asymptomatic and affected carriers regarding duplications (14% vs 23% and 20%, respectively), however because of comparatively small samples, these differences do not reach statistical significance.

**Table 1:** Sex ratio in carriers of mosaicism for segmental autosomal mosaicism

Study groups	Unbalanced rearrangements			Balanced rearrangements		
	Males	Females	Sex ratio	Males	Females	Sex ratio
Asymptomatic carriers of gonadal mosaicism (transmitting parents)^a^	7	28	0.25	9	8	
Asymptomatic carriers of somatic mosaicism (patients with reproductive disorders and fortitously detected carriers)^a^	2	8	0.25	15	14	
Total	9	36	0.25*	24	22	1.1
Affected carriers of somatic and gonadal mosaicism^a,b^	95	133	0.7	7	6	
Prenatally diagnosed affected carriers^c^	15	23	0.65	2	1	
Total	110	156	0.7**	9	7	1.3
Prenatally diagnosed normal carriers^c^	18	10	1.8 ***	12	8	1.5

^a^Kovaleva, Cotter, 2016; ^b^Kovaleva, Cotter, 2017; ^c^present data

*different from population ratio of 1.06, *p* < 0.0001

**different from population ratio of 1.06, *p* = 0.0009 (binomial test)

***different from SR = 0.7 in combined sample, *p* = 0.0348 (chisquare, Yates-corrected()

### Pregnancy outcomes

Clinical evaluation of the pregnancy outcomes was reported in 74 of 88 (84%) cases with mosaicism for unbalanced Rea. Apparently normal outcome was reported for 28 (38%) of cases. The proportion of apparently abnormal outcomes varied from 30% in carriers of duplications to 62% in carriers of unbalanced translocations, with an average incidence of 50%. For cases of balanced Rea, the outcome was reported in 27 of 33 (82%) cases. If we classify the three “liveborn” cases as a normal outcome, the apparently abnormal outcome was reported in 3 of 27 (11%) of the cases. Several cases not attributed to any category and the cases with no information on the outcome were included in the sample cohort.

### Proportion of cells with unbalanced Rea in amniocytes cultures from normal and affected carriers

The proportion of abnormal cells in amniocytes was reported in 57 cases. On average, in carriers with abnormal outcome (*n* = 33), the mean proportion of abnormal cells was 50.5%, and the corresponding figure for unaffected carriers (*n* = 24) was 26%. Since the number of tested cells was not specified in every case, a valid statistical analysis of the figures obtained was not possible. Therefore, we analyzed a number of individuals with a proportion of abnormal cells reported to be larger than 50%. Nineteen of thirty three (58%) carriers with abnormal outcome displayed a high proportion of amniocytes with abnormality compared to 6 of 24 (25%) carriers with normal outcome, the difference between these groups is statistically significant at *p* = 0.014. This data corroborates with the results of a previous study on carriers of somatic/gonadal segmental mosaicism where a high proportion of Rea cells detected in cultured T-lymphocytes was found to be associated with clinical manifestation of chromosomal imbalance [6]. This data is also consistent with previous reports showing a higher frequency of abnormal phenotypes with high frequencies of the abnormal cell lines in whole chromosome aneuploidy mosaicism [10, 11].

### Male to female ratio

More male than female carriers of unbalanced Rea were observed with an apparently normal outcome (18 of 28), SR = 1.8. In contrast, fewer male carriers for an apparently abnormal outcome were observed (15 of 37), SR = 0.73. Of interest, the mothers of normal males had a mean age of 38 year (lim 32 years - 42 years), whereas mothers of affected males had a mean age of 33.6 year (lim 26 years −38 years). These observations did not reach statistical significance because of the small number of published prenatally diagnosed cases with known sex and parental ages. Data from Table 1 shows a four-fold statistically significant deficit of males among asymptomatic carriers of mosaic unbalanced Rea (*p* < < 0.0001). Note that in all studied groups, there is a slight male prevalence evident among carriers of balanced Rea, not different statistically from the population ratio of 1.06. Affected carriers of somatic and gonadal mosaicism and prenatally diagnosed affected carriers, showed a similar male to female ratio. Combining both groups demonstrated a notable female prevalence, different statistically from the population ratio of 1.06, *p* = 0.0009. Comparing SR = 1.8 among carriers of mosaic unbalanced Rea presenting with normal outcome, with SR = 0.7 in the combined group of affected carriers, we determined a statistically significant difference between them at *p* = 0.0348.

Data on male to female ratio among carriers of mosaicism in spontaneous abortuses (7 males/23 females) should be considered with caution due to possible maternal cell contamination (MCC) [12]. The data in Additional file 7: Table S7 showed that at least 11 female cases were not associated with MCC. Assuming MCC as the cause of mosaicism in the remaining cases (which is unlikely), the male to female ratio among spontaneously aborted carriers of unbalanced N/Rea (7 males/11 females) still demonstrates absence of male predominance. This observation suggests that the female predominance in affected prenatally detected fetuses as a result of high male intrauterine death implausible.

Based on the data presented in this paper, we suggest a male-specific selection against chromosomal abnormalities which provides better prospects for male carrier fetuses. Several authors suggested that female embryos are relatively delayed in early embryonic development [13, 14]. The delay in early female development has been ascribed to the absence of a Y chromosome. However, the process of X inactivation, since it may occur when there are ≤ 10 cells in the embryo, might itself contribute to a slight delay in early female embryo development [13]. A higher male cell turnover might facilitate effective selection against abnormal cell line.

As noted, there is a paucity of prenatal N/Rea reports, given large-scale prenatal testing worldwide. We encourage the reporting of additional cases to further evaluate these observations.

## Conclusion

Previous reports have identified a deficit of male among asymptomatic carriers of N/unbalanced Rea. Three mechanisms were proposed for explaining this phenomenon, including a high intrauterine lethality of male carriers, a male-specific selection against abnormal cells in the early embryo development, or a high instability in the early female embryonic development. The current data suggests a male-specific selection against chromosomal abnormalities.

## Additional files

Additional file 1: Table S1. Mosaicism for deletions. Tabular data presenting details of carriers of mosaicism for deletion: karyotype, parental ages, proportion of abnormal cell line(s), confirmation testing, indications for cytogenetic testing, outcome of the pregnancy. (XLSX 8 kb)

Additional file 2: Table S2. Mosaicism for duplications. Tabular data presenting details of carriers of mosaicism for duplication: karyotype, parental ages, proportion of abnormal cell line(s), confirmation testing, indications for cytogenetic testing, outcome of the pregnancy. (XLSX 6 kb)

Additional file 3: Table S3. Mosaicism for ring chromosomes. Tabular data presenting details of carriers of mosaicism for ring chromosomes: karyotype, parental ages, proportion of abnormal cell line(s), confirmation testing, indications for cytogenetic testing, outcome of the pregnancy. (XLSX 6 kb)

Additional file 4: Table S4. Mosaicism for unbalanced translocations. Tabular data presenting details of carriers of mosaicism for unbalanced translocation: karyotype, parental ages, proportion of abnormal cell line(s), confirmation testing, indications for cytogenetic testing, outcome of the pregnancy. (XLSX 6 kb)

Additional file 5: Table S5. Mosaicism for other unbalanced rearrangements. Tabular data presenting details of carriers of mosaicism for other unbalanced rearrangement: karyotype, parental ages, proportion of abnormal cell line(s), confirmation testing, indications for cytogenetic testing, outcome of the pregnancy. (XLSX 6 kb)

Additional file 6: Table S6. Mosaicism for apparently balanced rearrangements. Tabular data presenting details of carriers of mosaicism for apparently balanced rearrangement: karyotype, parental ages, proportion of abnormal cell line(s), confirmation testing, indications for cytogenetic testing, outcome of the pregnancy. (XLSX 7 kb)

Additional file 7: Table S7. Mosaicism for structural rearrangements in miscarried/spontaneously aborted fetuses. Tabular data presenting details of miscarried/spontaneously aborted fetuses with structural rearrangement: karyotype, parental ages, proportion of abnormal cell line(s), reasons for cytogenetic testing, and probality of maternal cell contamination in female specimens. (XLSX 8 kb)

Additional file 8: Reference list for Tables S1-S7. (DOC 80 kb)
