# Supplementary material for: Mosaicism for structural non-centromeric autosomal rearrangement in prenatal diagnoses: evidence for sex-specific selection against chromosomal abnormalities
Source: Mol Cytogenet. 2017 Dec 11;10:45. doi: 10.1186/s13039-017-0346-0 (PMC5725842; doi:10.1186/s13039-017-0346-0)
Supplement: Supplementary file 8 — Reference list for Tables S1-S7. (DOC 80 kb) [file 13039_2017_346_MOESM8_ESM.doc]

Supplemental References

1. Adam LR, Simpson GF, Kalousek DK. 1993. Confined placental mosaicism (CPM) and pregnancy outcome – evaluation of three cases and review of literature. Am J Hum Genet 53(Suppl 3):1378.
2. Andrews T, Mould S, Fitchett M, Gregson NM, Millener R, Wallbanks G, Seabright M. Structural mosaicism – problems in prenatal diagnosis. J Med Genet. 1984;21:54.
3. Anselem O, Bazin A, Mechler C, Blin G, Garel C, Aboura A, et al. Prenatal Diagnosis of 18q-Syndrome: A Case of Fetal Mosaicism with a Normal Karyotype in Chorionic Villi. Fetal Diagn Ther. 2010;28:180-185.
4. Arakaki DT, Waxman SH. Chromosome abnormalities in early spontaneous abortions. J Med Genet. 1970;7:118-124.
5. Babu A, Popescu S, Bogosian V, Penchaszdeh VB. Prenatal detection of mosaic deletion of 16p, del(16)(p12), coincident with the expression of fragile site, fra(16)(p12), in amniotic fluid cells. Am J Hum Genet. 1995; 57(Suppl 4):1989.
6. Basaran N, Sener T, Durak B, Berkil H, Ozon YH. Prenatal diagnosis of the fetus with mosaic del(12)(q21.32q22). Eur J Hum Genet. 2001;8(Suppl):P0677.
7. Beck-Lippert W, Matt B, Muller-Navia J, Schleiermacher E. Prenatal diagnosis of a ring chromosome (20) – mosaic. Am J Hum Genet. 1991;49(suppl 4):275.
8. van den Berg C, Van Opstal D, Polak-Knook J, Galjaard RJ. (Potential) false-negative diagnoses in chorionic villi and a review of the literature. Prenat Diagn. 2006;26:401-408.
9. Di Bernardo C, Rinaldi R, Scassellati G, Pittalis MC, Grammatico B, Martinelli E, Grammatico P. Dup(11)(q13-qter) mosaicism in prenatal diagnosis: case report. Ann Genet. 2003;46(Suppl 2-3):216.
10. Bettio D, Venci A, Levi Setti PE. Chromosomal abnormalities in miscarriages after different assisted reproduction procedures. Placenta. 2008;29:S126-S128.
11. Bui TH, Iselius L, Lindsten J. European collaborative study on prenatal diagnosis: mosaicism, pseudomosaicism and single abnormal cells in amniotic fluid cell cultures. Prenat Diagn, 1984;4(Spec Issue):145-162.
12. Caron L, Tihy F, Dallaire L. Frequencies of chromosomal abnormalities at amniocentesis: over 20 years of cytogenetic analyses in one laboratory. Am J Med Genet. 1999;82:149-154.
13. Chen CP, Tzen CY, Chang TY, Lin CJ, Wang W, Lee CC, Chen LF, Chen WL. Prenatal diagnosis of de novo mosaic distal 18q deletion associated with congenital anomalies. Ultrasound Obstet Gynecol. 2003;21:202-204.
14. Chen H, Mirkin D, Yang S. De novo 17q paracentric inversion mosaicism in a patient with Beemer-Langer type short rib-polydactyly syndrome with special consideration to the classification of short rib polydactyly syndromes. Am J Med Genet. 1994;53:165-171.
15. Chen CP, Lee CC, Chang TY, Town DD, Wang W. Prenatal diagnosis of mosaic distal 5p deletion and review of the literature. Prenat Diagn. 2004;24:50-57.
16. Chen CP, Lee CC, Chang TY, Town DD, Wang W. Discrepancy between the fetus and extra-embryonic tissues in prenatally detected mosaic distal 5p deletion. Cenet Couns. 2004;15:473-476.
17. Chen CP, Chern SR, Lee CC, Lin SP, Chang TY, Wang W. Prenatal diagnosis of mosaic 22q11.2 microdeletion. Prenat Diagn. 2004;24:660-662.
18. Chen C-P, Chen Y-J, Chern S-R, Tsai F-J, Lin H-H, Lee C-C, Wang W. Prenatal diagnosis of mosaic 1q31.3q32.1 trisomy associated with occipital encephalocele. Prenat Diagn. 2008;28:865-867.
19. Chen C-P, Su Y-N, Hsu C-Y, Chern S-R, Lee C-C, Chen Y-T, et al. Mosaic deletion-duplication syndrome of chromosome 3: Prenatal molecular cytogenetic diagnosis using cultured and uncultured amniocytes and association with fetoplacental discrepancy. Taiwan J Obstet Gynecol. 2011;50:485e491.
20. Chen C-P, Kuo Y-T, Lin S-P, Su Y-N, Chen Y-J, Hsueh R-Y, et al. Mosaic ring chromosome 18, ring chromosome 18 duplication/deletion and disomy 18:  perinatal findings and molecular cytogenetic characterization by fluorescence in situ hybridization and array comparative genomic hybridization. Taiwan J Obstet Gynecol. 2010;49:327-332.
21. Christiansen LR, Lage JM, Wolff DJ, Shashidhar Ppai G., Harley RA. Mosaic duplication 1(q11q44) in an infant with nephroblastomatosis and mineralization of extraplacental membranes. Pediatr Develop Pathol. 2005;8:115-123.
22. Cotter PD, Babu A, Willner JP, Desnick RJ. Prenatal diagnosis and outcome of mosaicism for a de novo unbalanced translocation identified in amniocytes. Prenat Diagn. 1998;18:857-861.
23. Crandall BF, Lebherz TB, Rubinstein L, Robertson RD, Sample WF, Sarti D, Howard J. Chromosome findings in 2,500 second trimester amniocenteses. Am J Med Genet. 1980;5:345-356.
24. Descartes M, Baldwin L, Cosper P, Carroll A. Mosaicism for duplication 17q21-qter with lymphedema and normal phenotype. Genet Med. 1999;1(suppl):A68.
25. Deshpande M, Harper J, Holloway M, Palmer R, Wang R. Evaluation of array comparative genomic hybridization for genetic analysis of chorionic villus sampling from pregnancy loss in comparison to karyotyping and multiplex ligation-dependent probe amplification. Genet Test Mol Biomark. 2010;14:421-424.
26. Doria S, Carvalho F, Ramalho C, Lima V, Francisco T, Machado AP, et al. An efficient protocol for the detection of chromosomal abnormalities in spontabeous miscarriage and fetal death. Eur J Obstet Gynecol Reprod Biol. 2009;147:144-150.
27. Eckmann-Scholtz C, Mallek J, von Kaisenberg CS, Arnold NK, Jonat W, Reiner S, et al. Chromosomal mosaicism in prenatal diagnosis: correlation with first trimester screening and clinical outcome. J Perinat Med. 2012;40:215-223.
28. Eiben B, Leipold M, Schubbe I, Ulbrich R, Hansmann I. Partial deletion of 4p in fetal cells not presented in chorionic villi. Clin Genet. 1988;33:49-52.
29. Faivre L, Rousseau T, Laurent N, Gosset P, Sanlaville D, Thauvin-Robinet C, Prenatal overgrowth and mosaic trisomy 15q25-qter including the IGF1 receptor gene. Prenat Diagn. 2004;24:393-395.
30. [Featherstone T](http://www.ncbi.nlm.nih.gov/entrez/query.fcgi?db=pubmed&cmd=Search&term=), [Cheung SW](http://www.ncbi.nlm.nih.gov/entrez/query.fcgi?db=pubmed&cmd=Search&term=), [Spitznagel E](http://www.ncbi.nlm.nih.gov/entrez/query.fcgi?db=pubmed&cmd=Search&term=), [Peakman D](http://www.ncbi.nlm.nih.gov/entrez/query.fcgi?db=pubmed&cmd=Search&term=). Exclusion of chromosomal mosaicism in amniotic fluid cultures: determination of number of colonies needed for accurate analysis. Prenat Diagn. 1994;14:1009-1017.
31. Ferguson-Smith MA, Yates JR. Maternal age specific rates for chromosome aberrations and factors influencing them: report of a collaborative european study on 52 965 amniocenteses. Prenat Diagn. 1984;4(Spec Issue):5-44.
32. Fischer W, Dermitzel A, Osmers R, Pruggmayer M. Complete karyotype discrepancy between placental and fetal cells in a case of ring chromosome 18. Prenat Diagn. 2001;21:481-483.
33. Gardner RJ, Dockery HE, Fitzgerald PH, Parfitt RG, Romain DR, Scobie N, Shaw RL, Tumewu P, Watt AJ. Mosaicism with a normal cell line and an autosomal structural rearrangement. J Med Genet. 1994;31:108-114.
34. [Giardino](https://www.ncbi.nlm.nih.gov/pubmed/?term=Giardino D%5BAuthor%5D&cauthor=true&cauthor_uid=20939888) D, [Vignoli](https://www.ncbi.nlm.nih.gov/pubmed/?term=Vignoli A%5BAuthor%5D&cauthor=true&cauthor_uid=20939888) A, [Ballarati](https://www.ncbi.nlm.nih.gov/pubmed/?term=Ballarati L%5BAuthor%5D&cauthor=true&cauthor_uid=20939888) L, Recalcati MP, Russo S, Camporeale N, et al. Genetic investigations on 8 patients affected by ring 20 chromosome syndrome. [BMC Med Genet](https://www.ncbi.nlm.nih.gov/pmc/articles/PMC2967536/). 2010;1:146.
35. Gillessen-Kaesbach G, Ngo NT. Ring 19 mosaicism detected during prenatal diagnosis. Prenat Diagn. 1990;10:683-687.
36. [Glass IA](http://www.ncbi.nlm.nih.gov/pubmed/?term=Glass IA%5BAuthor%5D&cauthor=true&cauthor_uid=16267671), [Rauen KA](http://www.ncbi.nlm.nih.gov/pubmed/?term=Rauen KA%5BAuthor%5D&cauthor=true&cauthor_uid=16267671), [Chen E](http://www.ncbi.nlm.nih.gov/pubmed/?term=Chen E%5BAuthor%5D&cauthor=true&cauthor_uid=16267671), [Parkes J](http://www.ncbi.nlm.nih.gov/pubmed/?term=Parkes J%5BAuthor%5D&cauthor=true&cauthor_uid=16267671), [Alberston DG](http://www.ncbi.nlm.nih.gov/pubmed/?term=Alberston DG%5BAuthor%5D&cauthor=true&cauthor_uid=16267671), [Pinkel D](http://www.ncbi.nlm.nih.gov/pubmed/?term=Pinkel D%5BAuthor%5D&cauthor=true&cauthor_uid=16267671), [Cotter PD](http://www.ncbi.nlm.nih.gov/pubmed/?term=Cotter PD%5BAuthor%5D&cauthor=true&cauthor_uid=16267671). Ring chromosome 15: characterization by array CGH. [Hum Genet.](http://www.ncbi.nlm.nih.gov/pubmed/16267671) 2006;118:611-617.

| |  | | --- | |
| --- | --- |

| |  | | --- | |
| --- | --- |

| |  | | --- | |
| --- | --- |

1. [Golbus MS](http://www.ncbi.nlm.nih.gov/sites/entrez?Db=pubmed&Cmd=Search&Term=), [Epstein CJ](http://www.ncbi.nlm.nih.gov/sites/entrez?Db=pubmed&Cmd=Search&Term=), [Halbasch G](http://www.ncbi.nlm.nih.gov/sites/entrez?Db=pubmed&Cmd=Search&Term=), [Stephens JD](http://www.ncbi.nlm.nih.gov/sites/entrez?Db=pubmed&Cmd=Search&Term=), [Hall BD](http://www.ncbi.nlm.nih.gov/sites/entrez?Db=pubmed&Cmd=Search&Term=). Prenatal genetic diagnosis in 3000 amniocenteses. [N Engl J Med.](javascript:AL_get(this, 'jour', 'N Engl J Med.');) 1979;300:157-163.
2. Golden WL, Kirson L, Doty L, Wilson WG. Prenatal detection of a true mosaic for a balanced reciprocal translocation. Am J Hum Genet. 1991;49(Suppl):216A.
3. Gosden C, Nicolaides K H, Rodeck CH. Fetal blood sampling in investigation of chromosome mosaicism in amniotic fluid cell culture. Lancet. 1988;i:613-617.
4. [Grati FR](http://www.ncbi.nlm.nih.gov/entrez/query.fcgi?db=pubmed&cmd=Search&itool=pubmed_AbstractPlus&term=), [Grimi B](http://www.ncbi.nlm.nih.gov/entrez/query.fcgi?db=pubmed&cmd=Search&itool=pubmed_AbstractPlus&term=), [Frascoli G](http://www.ncbi.nlm.nih.gov/entrez/query.fcgi?db=pubmed&cmd=Search&itool=pubmed_AbstractPlus&term=), [Di Meco AM](http://www.ncbi.nlm.nih.gov/entrez/query.fcgi?db=pubmed&cmd=Search&itool=pubmed_AbstractPlus&term=), [Liuti R](http://www.ncbi.nlm.nih.gov/entrez/query.fcgi?db=pubmed&cmd=Search&itool=pubmed_AbstractPlus&term=), [Milani S](http://www.ncbi.nlm.nih.gov/entrez/query.fcgi?db=pubmed&cmd=Search&itool=pubmed_AbstractPlus&term=), et al. Confirmation of mosaicism and uniparental disomy in amniocytes, after detection of mosaic chromosome abnormalities in chorionic villi. Eur J Hum Genet. 2006;14:282-288.
5. Gunduz C, Alpman A, Karaca E, Cancaya T, Bora E, Sagol S, et al. Seven cases of chromosomal mosaicism detected in amniocentesis and karyotype, phenotype correlations. Eur J Hum Genet. 2002;9(Suppl):P0984.
6. Hastings RJ, Watson SG, Chitty LS. Prenatal finding of a fetus with mosaicism for two balanced de novo chromosome rearrangements. Prenat Diagn. 1999;19:77-80.
7. Hook EB, Cross PK. Rates of mutant and inherited structural cytogenetic abnormalities detected at amniocentesis: results on about 63.000 fetuses. Ann Hum Genet. 1987;51:27-55.
8. Howard PJ. Prenatal detection of a de novo duplication of the long arm of chromosome 7. Prenat Diagn. 1987;7:373-376.
9. Hsu LY, Yu MT, Richkind KE, Van Dyke DL, Crandall BF, Saxe DF, et al. Incidence and significance of chromosome mosaicism involving an autosomal structural abnormality diagnosed prenatally through amniocentesis: a collaborative study. Prenat Diagn. 1996;16:1-28.
10. Jenderny J. Chromosome aberrations in a large series of spontaneous miscarriages in the German population and review of the literature. Mol Cytogenet. 2014;7:38.
11. Jewell AF, Simpson GF, Pasztor L, Keene CL, Sullivan BA, Schwartz S. Prenatal diagnosis of two cases of de novo dup(12p), identified by fluorescence in situ hybridization (FISH). Am J Hum Genet. 1992;51(Suppl 4):81A.
12. Karaoguz MY, Biri A, Pala E, Kan D, Poyraz A, Kurdoglu M, Percin EF. A case with mosaic partial duplication of 1q: prenatal and postmortem clinical and cytogenetic evaluations. Genet Couns. 2006;17:197-204.
13. Kim ES, Ryu HM, Yang JH, Kim MY, Park SY, Choi SK, et al. Intrauterine growth retardation associated with confined placental mosaicism of ring chromosome 15. Am J Hum Genet. 1997;61(Suppl 4):A378.
14. King PA, Ghosh A, Tang M. Mosaic partial trisomy 17q2. J Med Genet. 1991;28:641-643.
15. Kriplani A, Banerjee N, Jobanputra V, Kulcheria K. Mosaic partial trisomy of chromosome 5(q33-qter) associated with fetal polycystic kidneys. Acta Genet Gemellol (Roma). 1998;47:125-129.
16. Lippman A, Tomkins DJ, Shime J, Hamerton JL. Canadian multicentre randomized clinical trial of chorion villus sampling and amniocentesis/ Final report. Prenat Diagn. 1992;12:385-476.
17. McGavran L, Berry RS, Manchester DK, Reiley T. Mosaic del(10)(q23) and FRA10A in a patient with microcephaly and myoclonic seizures. Am J Hum Genet. 1988;43(Suppl 3):A114.
18. Miguez L, Villa O, Santos M, Blasco V, Alegre M, Sostoa M,et al. Prenatal cytogenetic characterization of a mosaic partial trisomy 15q by G-banding, FISH, CGH and array-CGH. Chromosome Res. 2005 ;13(Suppl 1):137.
19. Mikkelsen M, Stene J. Previous child with Down syndrome and other chromosome aberrations. In: Murken J-D, Stengel-Rutkowski S, Schwinger E (eds). Prenatal diagnosis: Proc. 3rd Eur Conf on Prenatal Diagnosis of Genetic Disorders, Enke, Stuttgard. 1979:22-29.
20. Nagaishi M, Yamamoto T, Iinuma K, Shimomura K, Berend SA, Knops J. Chromosome abnormalities identified in 347 spontaneous abortions collected in Japan. J Obstet Gynaecol Res. 2004;30:237-241.
21. Ogasawara M, Aoki K, Okada S, Suzumori K. Embryonic karyotype of abortuses in relation to the number of previous miscarriages. Fertil Steril. 2000;73:300-304.
22. Pérez M, Gean E, Miro E, Degollada M, Plensa I, Aguayo A, Ojanguren I. Two cases of deletion, del(8)(p) mosaicism detected in amniocytes. Ann Genet. 2001;44(Suppl 1):S142.
23. Phelan MC, Brown EF, Rogers RC. Prenatal diagnosis of mosaicism for deletion 22q13.3. Prenat Diagn. 2001;21:1100-1102.
24. Pittalis MC, Mattarozzi A, Menozzi C, Malacarne M, Baccolini I, Farina A, et al. Structural chromosomal abnormalities detected during CVS analysis and their role in the prenatal ascertainment of cryptic subtelomere rearrangements. Am J Med Genet Part A. 2013;161A:2559-2563.
25. Porter S, Wilson E, Tyler X, Warren R, ffrench-Constant C, Pearson J. A case of discordant related abnormal karyotypes from chorionic villi and amniocytes. Prenat Diagn. 1999;19:887-890.
26. Qumsiyeh MB. Chromosome abnormalities in the placenta and spontaneous abortions. J Mat-Fetal Med. 1998;7:210-212.
27. Riegel M, Baumer A, Wisser J, Acherman J, Schinzel A. Prenatal diagnosis of mosaicism for a del (22)(q13). Prenat Diagn. 2000;20:76-79.
28. Rodriguez C, Cimaroli T, Sciorra L, Guzman E, Smulian J, Day-Salvatore D. Prenatal diagnosis of duplication 4 p mosaicism. Am J Hum Genet. 1999;65(Suppl 4):2085.
29. Sahin FI, Yilmaz Z, Uckuyu A, Ozalp O, Tarim E, Yanik F. Prenatal diagnosis of mosaicism identified in amniotic fluid cells cultures. Eur J Hum Genet. 2005;13(Suppl 1):164.
30. Sahlin E, Gustavsson P, Lieden A, Papadogiannakis N, Bjäreborn L, Pettersson K, Nordenskjöld M, Iwarsson E. Molecular and cytogenetic analysis in stillbirth: results from 481 consecutive cases. Fetal Diagn Ther. 2014; 36:326-332.
31. Schmidt R, Dowling PK. The incidence of structural chromosomal rearrangements and “true” mosaicism in 5000 consequtive amniotic fluids. Am J Hum Genet. 1983;35(Suppl 6):152A.
32. Shaham M, Beachman S, Vogel J, McGivans M, Carnahan A, Chaban PA, Searle BM. Mosaicism for balanced and unbalanced structural chromosome rearrangements. Am J Hum Genet. 1992;51(Suppl 4):A296.
33. Simoni G, Fraccaro M, Gimelli G, Maggi F, Dagna Bricarelli F. False-positive and false-negative findings on chorionic villus sampling. Prenat Diagn. 1987;7:671-672.
34. Smith G, Rauch L, Jones J, Gannon C, Stewart F. Prenatal diagnosis of mosaic del(13)(q13) in association with parieto-occipital encephalocoele and unilateral anopthalmos. J Med Genet. 2005;42(Suppl 1):S65.
35. Sundberg K, Lundsteen C, Philip J. Early filtration amniocentesis for further investigation of mosaicism diagnosed by chorionic villus sampling. Prenat. Diag. 1996;16:1121-1127.
36. Tharapel AT, Michaelis RC, Velagaleti GV. Chromosome duplications and deletions and their mechanism of origin. Cytogenet Cell Genet. 1999;85:285-290.
37. Thies U, Bartels I, von Beust G, Bink K, Hansmann I, Rehder H, et al. Prenatal diagnosis and fetopathological findings in a fetus with ring chromosome 18. Fetal Diagn Ther. 1998;13:315-320.
38. [Valduga M](http://www.ncbi.nlm.nih.gov/pubmed/?term=Valduga M%5BAuthor%5D&cauthor=true&cauthor_uid=17761465), [Cannard VL](http://www.ncbi.nlm.nih.gov/pubmed/?term=Cannard VL%5BAuthor%5D&cauthor=true&cauthor_uid=17761465), [Philippe C](http://www.ncbi.nlm.nih.gov/pubmed/?term=Philippe C%5BAuthor%5D&cauthor=true&cauthor_uid=17761465), [Romana S](http://www.ncbi.nlm.nih.gov/pubmed/?term=Romana S%5BAuthor%5D&cauthor=true&cauthor_uid=17761465), [Miton A](http://www.ncbi.nlm.nih.gov/pubmed/?term=Miton A%5BAuthor%5D&cauthor=true&cauthor_uid=17761465), [Droulle P](http://www.ncbi.nlm.nih.gov/pubmed/?term=Droulle P%5BAuthor%5D&cauthor=true&cauthor_uid=17761465), et al. Prenatal diagnosis of mosaicism for 11q terminal deletion. [Eur J Med Genet.](http://www.ncbi.nlm.nih.gov/pubmed/?term=eur+j+med+genet+2007+50+475) 2007;50(6):475-481. Doi:10.1016/j.emg.2007.06.002

| |  | | --- | |
| --- | --- |

1. Van Buggenhout G, Cooreman G, Thienpont L, Fryns JP. Early urethral obstruction sequence and trisomy of the long arm of chromosome 1. Ann Génét. 1995;38:106-107.
2. Veenma D, Beurskens N, Douben H, Eussen B, Noomen P, Govaerts L, et al. Comparable low-level mosaicism in affected and non affected of a complex CDH patient. PLoS ONE. 5:e15348.
3. Vockley J, Inserra J A, Breg W R, Yang-Feng T L: "Pseudomosaicism" for 4p- in amniotic fluid cell culture proven to be true mosaicism after birth. Am J Med Genet. 1991;39:81-83.
4. Warburton D, Stein Z, Kline J, Susser M. Chromosome abnormalities in spontaneous abortion: data from the New York City study. In; Porter IH, Hook EB, eds. Human embryonic and fetal death. New York: Academic Press: 1980: p. 261-287
5. Wax JR, Benn P, Steinfeld JD, Ingardia CJ. Prenatally diagnosed sacrococcygeal teratoma: a unique expression of trisomy 1q. Cancer Genet Cytogenet. 2000;117:84-86.
6. Wiedschwendter A, Riha K, Duba HC, Kreczy A, Marth C, Schwarzler P. Prenatal diagnosis of de novo mosaic deletion 13q associated with multiple abnormalities. Ultrasound Obstet Gynecol. 2002;19:396-399.
7. Wilson MG, Lin MS. Prenatal diagnosis of mosaicism for del(18)(q12.2q21.1) and a normal cell line. J Med Genet. 1988;25:635-636.
8. Wu T, Yin B, Zhu Y, Li G, Ye L, Chen C, et al. Molecular cytogenetic analysis of early spontaneous abortions conceived from varying assisted reproductive technology procedures. Mol Cytogenet. 2016;9:79.
9. Yee Ha, Bernier FP, Chernos J. Prenatal diagnosis of del(6p23) mosaicism and FRA6A: a case report and review of the literature. Am J Hum Genet. 2000; 67(Suppl 4):2350.
